# Special Issue “Pharmaceutical Residues in the Environment”

**DOI:** 10.3390/molecules25122941

**Published:** 2020-06-26

**Authors:** Jolanta Kumirska

**Affiliations:** Department of Environmental Analysis, Faculty of Chemistry, University of Gdansk, Wita Stwosza 63, 80-308 Gdansk, Poland; jolanta.kumirska@ug.edu.pl

**Keywords:** pharmaceutical residues, fate in the environment, fate in WWTPs, ecotoxicity, antibiotic resistance, development of methods, environmental risk assessment

Pharmaceuticals, due to their pseudo-persistence and biological activity as well as their extensive use in human and veterinary medicine, are a class of environmental contaminants that is of emerging concern. In contrast to some conventional pollutants, they are continuously delivered at low levels, which might give rise to toxicity even without high persistence rates. These chemicals are designed to have a specific physiological mode of action and frequently to resist inactivation before exerting their intended therapeutic effect. These features, among others, make pharmaceuticals responsible for bioaccumulation and toxic effects in aquatic and terrestrial ecosystems. It is extremely important to know how to remove them from the environment and/or how to perform their biological inactivation. Hence, the detection, determination and analysis of the fates of pharmaceuticals and their metabolites in different compartments of the environment are some of the main tasks of modern analytical and environmental chemistry. An important limitation of such studies is the availability of sufficiently sensitive and reliable analytical methods for determining the different pharmaceuticals present in trace amounts in such complex matrices. Although great advances have been made in their detection in aquatic matrices, there are limited analytical methodologies for the trace analysis of target and non-target pharmaceuticals in matrices such as soils, sediments or biota. There are still many gaps in robust data on their fate and behavior in the environment, as well as on their threats to ecological and human health. This Special Issue has included nine current research and three review articles in this field.

Seven research articles deal with the presence of pharmaceuticals in wastewater samples and evaluate their fate, ecotoxicity and/or elimination in wastewater treatment plants (WWTPs) equipped with different purification technologies [1,2,3,4,5,6,7]. 

Giebułtowicz et al. [1] have provided a comprehensive overview of the presence of 26 selected antibiotics in two Polish WWTPs (wastewater and sludge) and have provided crucial information on their removal efficiency and their risk to resistance selection as well as cyanobacteria and eukaryotic species. They established that the removal efficiency of these compounds was more than 50% for both WWTPs. The highest antimicrobial resistance risk was estimated in the influents of WWTPs for azithromycin, ciprofloxacin, clarithromycin, metronidazole and trimethoprim and in the sludge samples for azithromycin, ciprofloxacin, clarithromycin, norfloxacin, trimethoprim, ofloxacin and tetracycline. 

Guedes–Alonso et al. [2] have studied the removal efficiencies of 11 pharmaceuticals in three wastewaters treated by conventional or natural purification systems over two years in order to determine the occurrence and removal of pharmaceutical residues in Gran Canaria (Spain). A combination of secondary treatments and reverse osmosis presents favorable removal efficiencies (over 95% for most studied compounds). However, all the target pharmaceuticals were present in the effluent samples. 

Zhang et al. [3] have found that constructed wetlands (CWs) could achieve a high removal efficiency of sulfamethoxazole (SMX) (>98%) and that the concentration of SMX in the bottom layer was higher compared with that in the surface layer. A degradation mechanism of SMX was also proposed. Moreover, the relative abundance of *sul* genes exhibited an increase, which tended to be stable throughout the treatment duration. 

The effectiveness of CWs for the removal of 15 pharmaceuticals and endocrine disrupting compounds in municipal WWTPs was also investigated by Wolecki et al. [4]. For the first time in such a study, three plants, namely *Cyperus papyrus* (Papyrus), *Lysimachia nemorum* (Yellow pimpernel) and *Euonymus europaeus* (European spindle), were taken into account. The investigation was performed using real municipal WWTP conditions and with the determination of target compounds not only in raw and treated wastewater but also in plant materials (a new ASE-SPE-GC-MS(SIM) method for this purpose was developed and validated in this study). The authors confirmed that the elimination efficiency of the investigated compounds from wastewater was in the range of 35.8% to above 99%. Moreover, *Lysimachia nemorum* was the most effective for the uptake of target compounds among the tested plant species. 

Nałęcz–Jawecki et al. [5] have evaluated the biological activity of four antidepressants, fluoxetine, sertraline, paroxetine and mianserin, on the ciliated protozoan *S. ambiguum.* Acute toxicity, bioconcentration and biotransformation studies were performed. The authors observed that sertraline was the most toxic among the studied antidepressants. However, the toxic effects occurred at concentrations at least two orders of magnitude higher than those determined in effluents and freshwaters. 

The main aim of the Pazda et al. research article [6] was to compare the occurrence of selected tetracycline- and sulfonamide resistance genes in raw influent and final effluent samples from two Polish WWTPs which were different in terms of size and applied biological wastewater treatment processes (conventional activated sludge (AS)-based in one WWTP and a combined conventional AS-based method with constructed wetlands (CWs) in the other). The genes selected for the study are commonly detected in wastewater samples, represent different resistance mechanisms, and are also located in different genetic elements (especially in mobile genetic elements which significantly influence the spread of antibiotic resistance genes (ARGs)). Furthermore, a method for the isolation of total DNA and the identification of selected ARGs in wastewater samples was developed. All thirteen ARGs coding resistance to tetracyclines, *tet (A, B, C, G, K, L, M, O, Q, X)* and sulfonamides *(sulI, sulII, sulIII)*, were detected in raw influent and final effluent samples from both WWTPs. The results of the comparative quantitative qPCR-based analyses in most cases showed the enrichment of the selected ARGs after the wastewater treatment processes (more than a 10-fold increase in five of the studied resistance genes was observed in the final effluent of a conventional WWTP). The results of this research article allowed the authors to estimate the scale of ARG spread in the environment, depending on the size and type of WWTP system, and highlight the need to implement high-efficiency preventive actions.

Pieczyńska et al. [7] have investigated the degradation of cytostatic drugs (CD), 5-Fuorouracil (5-FU), cyclophosphamide (CP) and ifosfamide (IF) and their mixtures, using an electrochemical filter press cell divided by a Nafion membrane in batch treatment (flow recirculation). The order of the CD degradation rate in single drug solutions and in mixtures was found to be 5-FU < CP < IF. The fundamental reaction mechanism, as well as the effects of natural water constituents on the kinetics and mechanisms of electrochemical oxidation of cytostatic drugs in their mixtures, were studied.

Two research articles describe the occurrence of pharmaceuticals in soil samples and evaluate their mobility and toxicity on environmental organisms [8,9].

Wychodnik et al. [8] established the influence of mass breeding of hens on soil contamination with 26 pharmaceuticals and caffeine (CAF). The results showed that the observed changes in pharmaceutical presence in the analyzed soil samples could be defined as seasonal (in all summer samples, less substances (four pharmaceuticals) were determined in contrast with samples collected in March 2019 (10 pharmaceuticals and CAF)). Moreover, concentration levels of sulfamethazine and sulfanilamide in samples collected in July 2019 were approximately five times higher than those collected in March 2019. The antibiotic resistance of 85 random bacterial strains isolated from soil samples was also determined. The level of bacterial resistance to antibiotics did not differ between the samples from intensive breeding farm surroundings and those from the reference area. 

The soil behavior of the veterinary drugs, lincomycin, monensin and roxarsone, and their toxicity on environmental organisms (algae, plants, daphnia, fish, earthworms and quails) have been investigated by Li et al. [9]. Lincomycin and roxarsone were characterized by moderate soil mobility; however, roxarsone’s ecotoxicity implied that it is a potential ecological risk. Monensin was the most toxic among the three drugs tested, and its higher affinity for soil made it easier to be accumulated.

Apart from research papers, three interesting review articles [10,11,12] have been published in this Special Issue. Two of them were written by Pereira et al. [10,11]. The first [10] tackled the source, fate and occurrence of 22 pharmaceuticals, six metabolites and transformation products belonging to seven therapeutic groups, in several aquatic compartments (wastewater influents and effluents, surface waters, groundwaters, seawaters, mineral waters and drinking waters). The second article [11] presents the issues of toxicity and the risk assessment of pharmaceuticals, using the occurrence data obtained in the first paper, highlighting and updating the current knowledge on this subject. Such an approach led to the integration of all of these issues under the scope of the environmental risk assessment, providing new and updated data not only on occurrence and toxicity but also relating to the risk assessment of human pharmaceuticals in the most important water compartments.

The last paper of this Special Issue, written by Treder et al. [12], deals with the use of ionic liquids (ILs) in liquid chromatography, gas chromatography and capillary electrophoresis for the determination of pharmaceuticals in environmental and biological matrices. Based on the large number of reported references, these compounds are very effective for the analysis of different classes of compounds. In addition, they are eco-friendly and therefore very useful in the analytical and preparative fields. However, the limitations that appear during their use show that success in experiments is not easy and this field of research requires further development.

In conclusion, the frequent detection of many pharmaceuticals in the environment has been an increasing concern due to their potential to cause undesirable ecological effects, which may range from endocrine disruption in fish and wildlife to antibiotic resistance in pathogenic bacteria. 
